# Association among Dietary Flavonoids, Flavonoid Subclasses and Ovarian Cancer Risk: A Meta-Analysis

**DOI:** 10.1371/journal.pone.0151134

**Published:** 2016-03-09

**Authors:** Xiaoli Hua, Lili Yu, Ruxu You, Yu Yang, Jing Liao, Dongsheng Chen, Lixiu Yu

**Affiliations:** 1 Department of Pharmacy, Union Hospital, Tongji Medical College, Huazhong University of Science and Technology, Wuhan, 430022, PR China; 2 Department of Obstetrics and Gynecology, Union Hospital, Tongji Medical College, Huazhong University of Science and Technology, Wuhan, 430022, PR China; National Cancer Center, JAPAN

## Abstract

**Background:**

Previous studies have indicated that intake of dietary flavonoids or flavonoid subclasses is associated with the ovarian cancer risk, but presented controversial results. Therefore, we conducted a meta-analysis to derive a more precise estimation of these associations.

**Methods:**

We performed a search in PubMed, Google Scholar and ISI Web of Science from their inception to April 25, 2015 to select studies on the association among dietary flavonoids, flavonoid subclasses and ovarian cancer risk. The information was extracted by two independent authors. We assessed the heterogeneity, sensitivity, publication bias and quality of the articles. A random-effects model was used to calculate the pooled risk estimates.

**Results:**

Five cohort studies and seven case-control studies were included in the final meta-analysis. We observed that intake of dietary flavonoids can decrease ovarian cancer risk, which was demonstrated by pooled *RR* (*RR* = 0.82, 95% CI = 0.68–0.98). In a subgroup analysis by flavonoid subtypes, the ovarian cancer risk was also decreased for isoflavones (*RR* = 0.67, 95% CI = 0.50–0.92) and flavonols (*RR* = 0.68, 95% CI = 0.58–0.80). While there was no compelling evidence that consumption of flavones (*RR* = 0.86, 95% CI = 0.71–1.03) could decrease ovarian cancer risk, which revealed part sources of heterogeneity. The sensitivity analysis indicated stable results, and no publication bias was observed based on the results of Funnel plot analysis and Egger’s test (*p* = 0.26).

**Conclusions:**

This meta-analysis suggested that consumption of dietary flavonoids and subtypes (isoflavones, flavonols) has a protective effect against ovarian cancer with a reduced risk of ovarian cancer except for flavones consumption. Nevertheless, further investigations on a larger population covering more flavonoid subclasses are warranted.

## Introduction

Ovarian cancer remains a highly lethal malignancy in the world, which has been a serious risk factor of health and safety for women, and a majority of patients are diagnosed in late stages of this disease [1–2]. As the poor prognosis for this disease, efforts to identify modifiable risk factors to reduce the risk of it are warranted. According to recent guidelines for cancer prevention published by the American Cancer Society Guidelines, diet remains one of the key lifestyle factors thought to modify cancer risk [3], although specific associations with the risk of ovarian cancer are less convincing [4].

**Fig 1:**
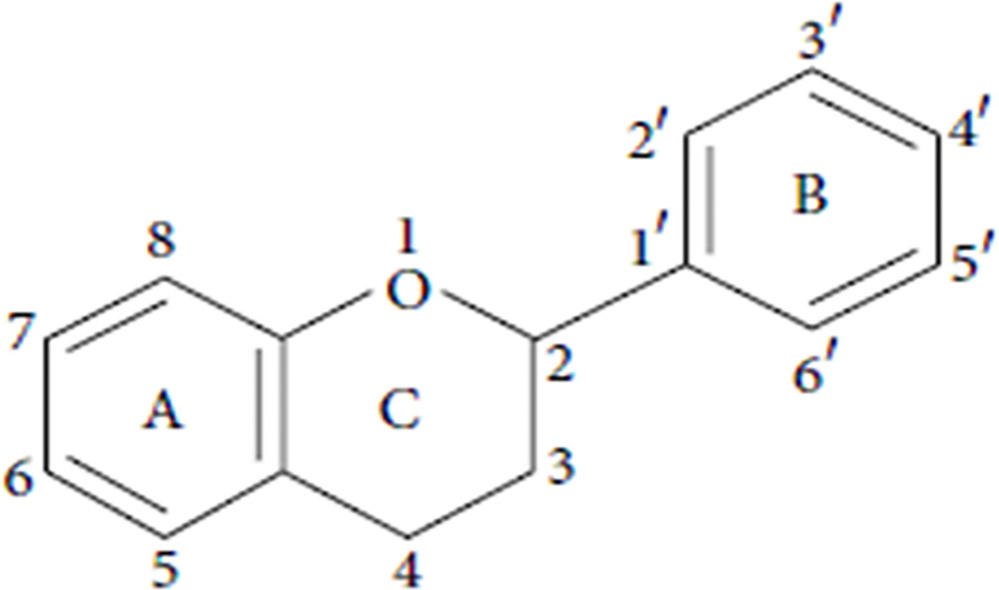
The basic structure of flavonoids.

Flavonoids with several functions in different physiological and pathological processes of cancer are polyphenolic compounds having a basic benzo-γ-pyrone structure (see Fig 1), which are widely distributed in all foods of plant origin such as fruit, vegetable, tea and wine [5–7]. Studies have suggested that consumption of high doses of dietary flavonoids has favorable health effects with a reduced risk of cancer such as those of the breast [8, 9], rectum [10], lung [11] and ovarian cancer [12]. Moreover, studies have demonstrated that the different pharmacological activities of dietary flavonoids on ovarian cancer depend on their structure [13]. Based on the range and structural complexity, dietary flavonoids in all food of plant can be categorized into six major subclasses as followed: flavones, isoflavones, flavonol, flavanones, anthocyanidins and flavan-3-ols [14,15], of which flavones, isoflavones and flavonols are reported in the highest amounts of consumption in the human diet and have biological activity on ovarian cancer [16–20]. Therefore, in this study we mainly focused on the association among total flavonoids, flavonoid subclasses (flavones, isoflavones and flavonols) and ovarian cancer risk.

Epidemiological studies and clinical trials have explored that flavonoids intake has the chemopreventive effects on carcinogenesis [21–23], and the adverse effects of flavonoids in human health are rare. Thus the anticancer activity of dietary flavonoids has become an upsurging research interest in the therapeutic and preventive potential. Considering intake of dietary flavonoids may reduce the risk of ovarian cancer, a number of studies have explored the association between dietary flavonoids and ovarian cancer risk. However, individual studies have yielded inconsistent or controversial findings. In addition, even though there was a meta-analysis which only analyzed the relationship between isoflavones (one subclass of dietary flavonoids) and the risk of ovarian cancer [24]. On the contrary, there was a systematic review of the relationships between dietary intake and ovarian cancer risk [25], which evaluated the role of all dietary intakes in ovarian cancer risk. To shed light on these conflicting results and to more precisely evaluate the association among dietary flavonoids, flavonoid subclasses and the risk of ovarian cancer for the guidance of clinical practice and prevention of ovarian cancer, we performed a meta-analysis of epidemiologic studies to investigate the association among dietary flavonoids, flavonoid subclasses and ovarian cancer risk.

## Material and Methods

### Search Strategy

We did our best to conduct a systematic literature search in PubMed, Google Scholar and ISI Web of Science up to April 25, 2015, without language restriction, regarding the association among dietary flavonoids, flavonoid subclasses and ovarian cancer risk. The search terms were as follows: (flavonoids or flavones or isoflavones or flavonols or flavanones or anthocyanidins or flavan-3-ols or polyphenolic compounds) and (Ovarian Neoplasms or Ovary Neoplasms or Ovarian Cancers or Ovary Cancers or Cancer of Ovarian or Cancer of Ovary or Ovarian tumor or Ovary tumor or Ovarian carcinoma or Ovary carcinoma). Furthermore, we performed a manual search by reviewing the related reference articles to identify any studies that were not identified from above literature searches. Only full length original journal articles were considered and articles have only abstracts or unpublished were excluded in this study. Our meta-analysis meta-analysis was conducted following the Meta-analysis of Observational Studies in Epidemiology (MOOSE) guidelines [26](PRISMA checklist reported in S1 Table).

### Inclusion and exclusion criteria

Studies were considered eligible if they met all of the following criteria: (1) the original articles described a case-control, cohort or randomized control design; (2) the article had either dietary flavonoids or subclasses of flavonoids intake as the exposure of interest; (3) the article reported the risk of ovarian cancers; and (4) the article reported 95% confidence intervals (CIs) with adjusted odds ratios (*OR*s) or relative risks (*RR*s) for ovarian cancer risk in subjects with the highest dietary flavonoids intake compared with those with the lowest dietary flavonoids intake. If multiple articles reported the risk of ovarian cancer from the same data, the most recently published data were selected, the overlapped cases but the latest were excluded. Meanwhile, if articles reported as an abstract, summary, comment letter, review or editorial, were also excluded.

### Data collection

On the basis of the inclusion and exclusion criteria listed above, the two independent investigators (LX Yu and XL Hua) extracted the following data: first author, publication year, study region, study design, data acquisition approach, number of cases and controls, types and consumption of flavonoids, controlled confounders adjusted for in multivariate analysis, *OR* or *RR* and 95% CI. We also assessed the quality of each study by using the Newcastle-Ottawa Scale (NOS) quality assessment criteria [27]. The quality scores of the studies ranged from 0 to 9.0, Scores<7.0 indicates low quality, otherwise indicates high quality.

### Statistical analysis

Statistical analyses were performed by using Stata 12.0 software (StataCorp LP, College Station, TX). *RR* with 95% CI was assessed for determining the associations between dietary flavonoids, flavonoid subclasses and ovarian cancer risk. The pooled *RR* were computed by the adjusted *RR*s or *OR*s and 95% CIs reported in the studies. The *OR*s were considered to correspond to *RR*s. Cochran *Q* statistic and *I*^2^ were used for the assessment of heterogeneity across the studies [28]. Nevertherless, in view of the limitations of Cochran Q, especially for small meta-analysis [29], *Tau*^2^ was also provided. In addition, a random effects model described by DerSimonian-Laird method was preferred to calculate the summarized estimates and corresponding 95% CIs [30]. Sensitivity analysis was performed to evaluate the robustness of the results of the combined effects, which were performed by sequential removal of each study. As publication biases in meta-analysis are more likely to affect small studies [31], just when the number of studies was more than10, Funnel plots and Egger’s linear regression tests were used to evaluate the publication bias (*p* < 0.05 suggested statistical significance) [32]. To explore the source of heterogeneity among the studies, subgroup analysis by flavonoid subclasses was performed.

## Results

### Literature search and Characteristics of the Included Studies

**Fig 2:**
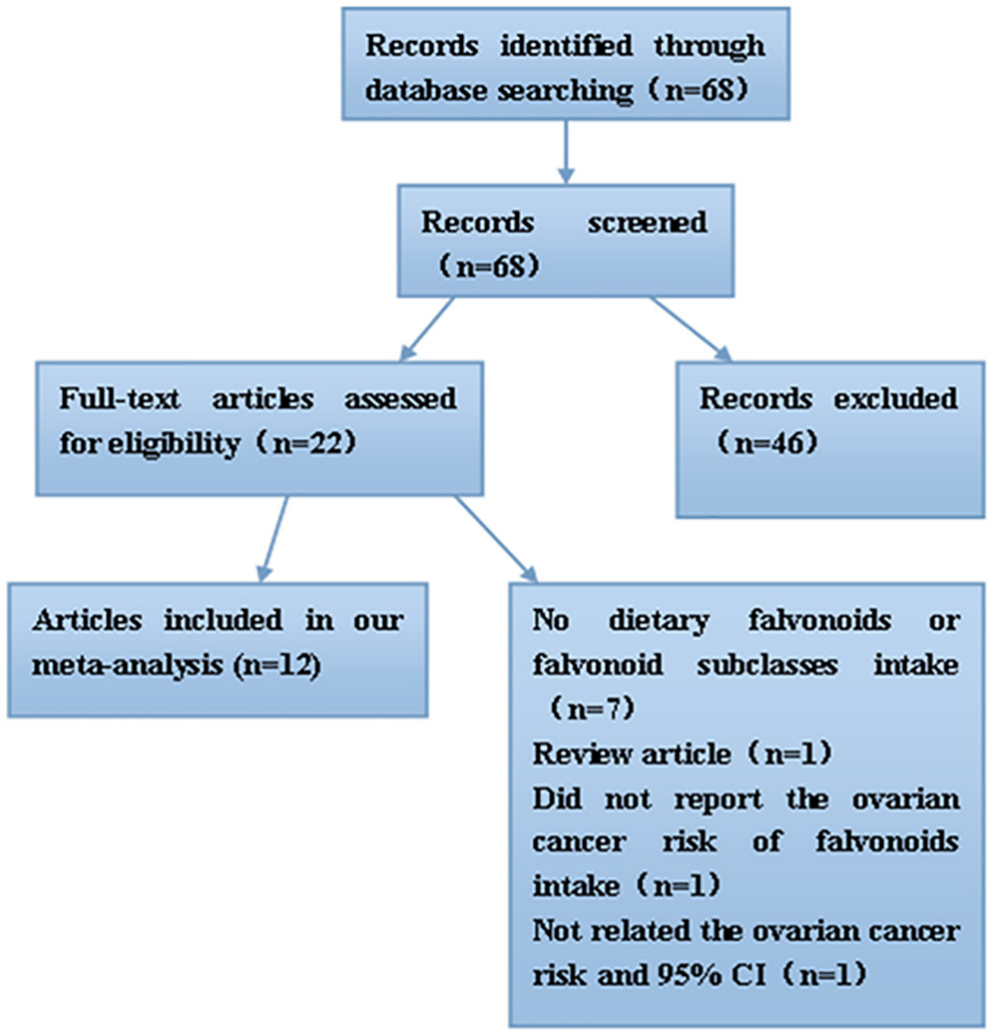
Flow chart of literature search and selection procedures on flavonoids and flavonoid subclasses in relation to the risk of ovarian cancer.

As shown in Fig 2, a total of 68 articles were identified in the initial search. Of these articles, 46 were excluded after reviewing the titles and abstracts, removing duplicates. Then, by thoroughly reading the full text 10 articles were also excluded because they did not provide information about flavonoids or flavonoid subclasses intake, ovarian cancer risk, or 95% CI. Finally, a total of 12 articles [12, 16–20, 33–38] with 6275 cases and 393776 controls met the inclusion criteria and were included in the final meta-analysis. The characteristics of the included studies were presented in Table 1, of which nine were high-quality studies (scores≥7.0). The selected studies were published between 2003 and 2014 spanning 11 years, and all of them were published in English. Among these 12 studies, 5 were prospective cohort studies, 7 were case-control studies, including 5 population based case-control studies, and 2 hospital based case-control studies. Moreover, 6 studies were from USA, 2 from China, and the rest were respectively from United Kingdom, Swedish, Australian, and Italy. The exposure assessments of flavonoids and flavonoid subclasses in 12 studies were made by questionnaire or published food composition data bases.

**Table 1:** Study characteristics of the association between dietary flavonoids, flavonoid subclasses and ovarian cancer risk in this meta-analysis. Note: FFQ: food frequency questionnaire; SFFQ: semi-quantitative food intake questionnaire; NOS: Newcastle-Ottawa Scale; BMI: body mass index.

Fisrt author, publicatin year and study region	Study design, data acquisition approach	Cases/ controls	Models	Types of flavonoids, flavonoid subclasses and consumption (low vs high) (mg/d)	*RR* or *OR* (95%CI)	Adjustments	Scores
Aedin Cassidy, 2014, United Kingdom	Prospective cohort, FFQ	723/171940	Cox proportional hazards models	total flavonoids (117.1 vs 713.4)	0.85 (0.66–1.09)	age, quintile of cumulative updated, energy-adjusted lactose intake and cumulative updated total energy intake, parity, the current questionnaire cycle, menopausal status, prmenopausal status, duration of oral contraceptive use, et al.	8
				flavones (0.7 vs 3.2)	0.87 (0.68–1.11)		
				flavonols (7.4 vs 30.2)	0.76 (0.59–0.98)		
				flavanones (7.8 vs 75.8)	0.79 (0.63–1.00)		
				flavan-3-ols (9.3 vs 133.7)	0.91 (0.71–1.16)		
				anthocyanin (2.5 vs 23.9)	0.95 (0.75–1.21)		
				proanthocyanin (54.0 vs 196.8)	0.92 (0.73–1.16)		
Maria Hedelin, 2011, Swedish	Prospective cohort, FFQ	163/47140	Cox proportional hazards models	total isoflavonoids (0.0005 vs 0.038)	1.15 (0.74–1.81)	age, oral contraceptives, age at menarche, parity, hormone replacement therapy, and intake of total energy intake, et al.	7
Lu Wang, 2009, USA	Prospective cohort, SFFQ	141/3234	Cox regression models	total quantified flavonoid (8.88 vs 47.44)	1.09 (0.60–2.01)	age, race, total energy intake and andomized treatment assignment, physical activity, postmenopausal status, et al.	7
Ellen T. Chang, 2007, USA	Prospective cohort, questionnaire	280/97275	Multivariable Cox proportional hazards regression	total isoflavonoids (117.1 vs 713.4)	0.56 (0.33–0.96)	race, total energy intake, parity, oral contraceptive use, strenuous exercise, wine consumption, and menopausal status et al.	6
				genistein (0.3 vs 1.1)	0.65 (0.42–1.02)		
				daidzein (0.3 vs 0.9)	0.75 (0.49–1.16)		
Margaret A. Gates, 2007, USA	Prospective cohort, SFFQ	347/66940	Cox proportional hazards models	total flavonoids (8.5 vs 42.6)	0.75 (0.51–1.09)	age, oral contraceptive use, parity, tubal ligation, smoking status, postmenopausal hormone use, physical activity, cumulative updated total energy intake, et al.	7
				myricetin (0.1 vs 2.4)	0.72 (0.50–1.04)		
				kaempferol (0.8 vs 11)	0.60 (0.42–0.87)	-	
				quercetin (6.3 vs 30.7)	0.80 (0.55–1.16)		
				luteolin (0.01 vs 0.07)	0.66 (0.49–0.91)		
				apigenin (0.2 vs 1.3)	1.33 (0.96–1.83)		
Andy H. Lee, 2014, China	Hospital based case-control, SFFQ	500/500	Unconditional logistic regression	isoflavones (26.7 vs 41.0)	0.45 (0.29–0.59)	age, BMI, physical activity, total energy intake, parity, oral contraceptive use, hormone replacement therapy, menopausal status, education, et al.	8
				daidzein (10.2 vs 16.9)	0.41 (0.29–0.59)		
				genistein (12.3 vs 21.1)	0.42 (0.30–0.60)		
				glycitein (1.9 vs 3.3)	0.38 (0.27–0.55)		
Annette S. Neill, 2014, Australian	Population based case-control, FFQ and published food composition data bases	1366/1414	Unconditional logistic regression	isoflavones (0.28 vs 4)	1.06 (0.79–1.43)	age, energy intake, age at menarche, parity, oral contraceptive use, hormone replacement therapy use, BMI, et al.	8
				daidzein (0.09 vs 1.2)	1.07 (0.8–1.43)		
				genistein (0.15 vs 2.7)	1.10 (0.82–1.48)		
				glycitein (0.02 vs 0.25)	0.93 (0.67–1.29)		
				formononetin (0.003vs 0.005)	0.97 (0.72–1.31)		
				biochanin A (0.015 vs 0.03)	1.09 (0.81–1.47)		
Elisa V. Bandera, 2011, USA	Population based case-control, FFQ	205/391	Unconditional logistic regression	total isoflavones (0.07 vs 0.41)	0.78 (0.48–1.27)	age, education, race, age at menarche, menopausal status, parity, oral contraceptive use, hormone replacement therapy use, BMI, et al.	7
				daidzein (0.02 vs 0.14)	0.8 (0.48–1.31)		
				glycitein (0.002 vs 0.0092l)	0.74 (0.46–1.21)		
				genistein (0.04 vs 0.25)	0.75 (0.46–1.23)		
				formononetin (0.0039 vs 0.0068)	0.69 (0.42–1.14)		
Margaret A. Gates, 2009, USA	Population based case-control, FFQ	1141/1183	Unconditional logistic regression	total flavonoids (0.9 vs 95)	1.06 (0.78–1.45)	age in years, study center, duration of oral contraceptive use, parity, history of tubal ligation, physical activity, total duration of breastfeeding, dietary intake of carotenoids, fiber intake, et al.	8
				myricetin (0.4 vs 2.8)	1.12 (0.85–1.49)		
				kaempferol (0.5 vs 6.9)	0.98 (0.73–1.32)		
				quercetin (3.5 vs 16.5)	1.14 (0.84–1.56)		
				luteolin (0.3 vs 2.9)	1.01 (0.58–1.74)		
				apigenin (0.03 vs 0.7)	0.79 (0.59–1.06)		
Marta Rossi, 2008, Italy	Hospital based case-control, FFQ	1031/2411	Logistic regression models	flavan-3-ols (16.3 vs 77.0)	0.89 (0.67–1.17)	age, study center, education, year of interview, parity, oral contraceptive use and family history of ovarian or breast cancer or both in first-degree relatives	8
				flavanones (12.2 vs 67.0)	1.28 (0.98–1.68)		
				flavonols (11.6 vs 28.8)	0.63 (0.47–0.84)		
				flavones (0.3 vs 0.7)	0.99 (0.76–1.29)		
				anthocyanidins (3.5 vs 19.4)	0.79 (0.60–1.04)		
				isoflavones (0.0128 vs 0.0325)	0.51 (0.37–0.69)		
				total flavonoids (67.3 vs 173.6)	1.07 (0.82–1.40)		
Min Zhang, 2004, China	Population based case-control, FFQ	254/652	Multivariate logistic regression	total isoflavonoids (11.6 vs 32.8)	0.51 (0.31–0.85)	age at diagnosis, education, area of residence, BMI, tobacco smoking, alcohol consumption, tea drinking, physical activity, parity, menopausal status, et al.	6
				daidzein (5 vs 14.9)	0.52 (0.31–0.87)		
				genistein (6.6 vs 20.9)	0.5 (0.30–0.84)		
				glycitein (0.4 vs 1.7)	0.59 (0.35–0.97)		
Susan E. McCan, 2003, USA	Population based case-control, FFQ	124/696	Unconditional logistic Regression	quercetin (10.16 vs 31.71)	0.71 (0.38–1.32)	age, education, total months menstruating, difficulty becoming pregnant, oral contraceptive use, menopausal status, et al.	6
				kaempferol (2.09 vs 8.57)	0.73 (0.39–1.34)		

Most individual studies were adjusted for a wide range of potential confounders, including age, education, cumulative updated total energy intake, BMI, physical activity, duration of oral contraceptive use, and history of postmenopausal hormone use.

### Meta-analysis results

**Fig 3:**
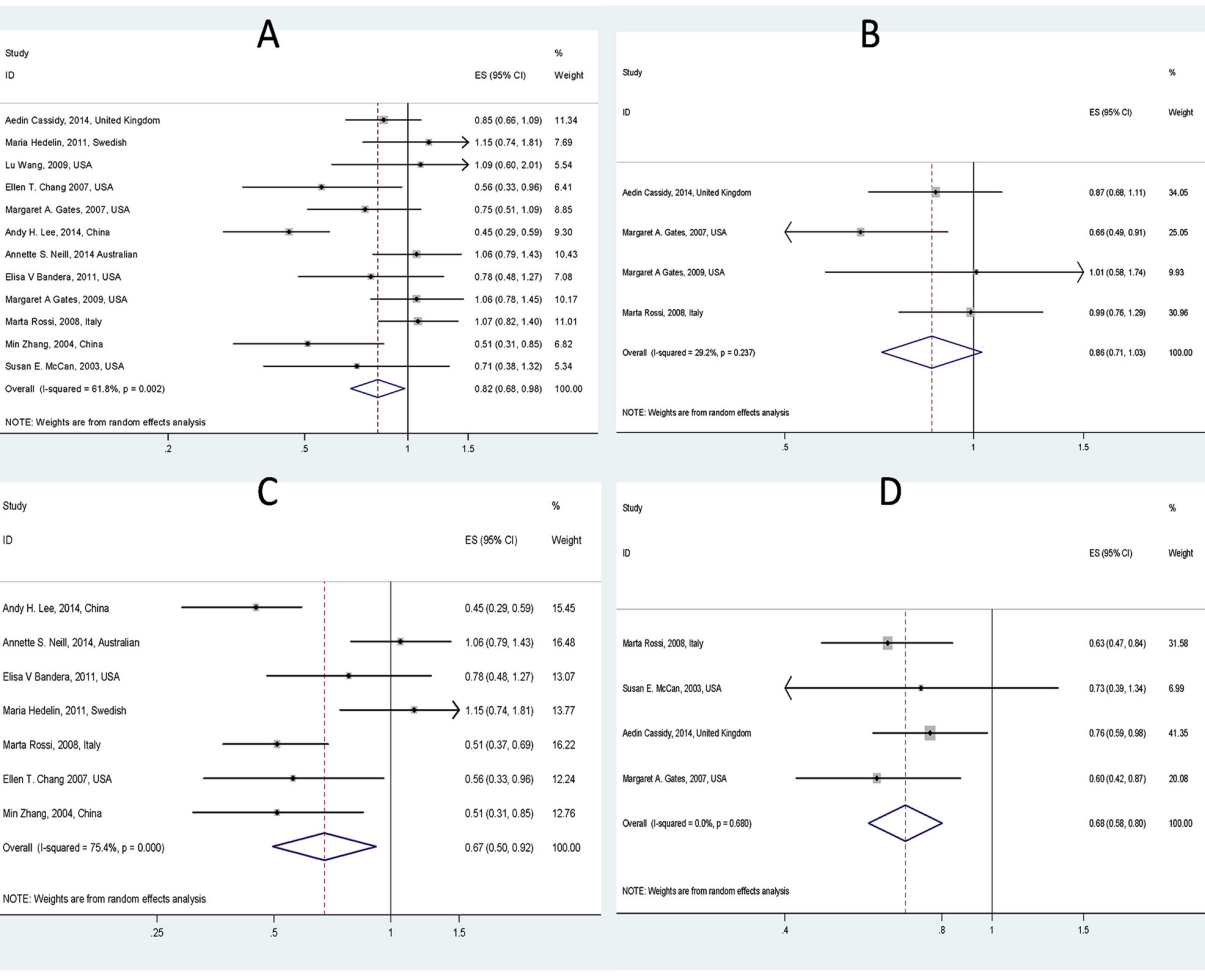
Forest plot of the RR with 95%CI for flavonoids, flavonoid subclasses intake and ovarian cancer risk (A. Total flavonoids, B. Flavones, C. Isoflavones and D. Flavonols).

The meta-analysis of the five cohort studies and seven case-control studies indicated that ovarian cancer risk was significantly reduced (*RR* = 0.82, 95% CI = 0.68–0.98) in women with highest intakes of total flavonoids, compared with that in those with lowest intakes of total flavonoids. That is, consumption of dietary flavonoids has a protective effect against ovarian cancer. The heterogeneity among the studies was significant (*I*^2^ = 62% (95%CI = 28%–80%), *Tau*^2^ = 0.059, *p* = 0.002) from a random effect model (Fig 3A).

### Subgroup meta-analysis

A subgroup meta-analysis was performed according to the subclasses of dietary flavonoids. We identified 4 studies about flavones intake and ovarian cancer risk, 4 studies about flavonols, and 7 studies about isoflavones. Overall, there was no sufficient evidence showing the association between intake of flavones and the ovarian cancer risk (*RR* = 0.86, 95% CI: 0.71–1.03) (Fig 3B). On the other hand, the incidence of ovarian cancer was decreased by consumption of isoflavones (*RR* = 0.67, 95% CI: 0.50–0.92) (Fig 3C) and flavonols (*RR* = 0.68, 95% CI: 0.58–0.80) (Fig 3D). Furthermore, in subgroup the heterogeneity was not significant for flavonols (*I*^2^ = 0.0% (95%CI = 0–85%), *Tau*^2^ = 0.0, *p* = 0.68) and flavones (*I*^2^ = 29.2% (95%CI = 0–74%), *Tau*^2^ = 0.01, *p* = 0.237) except for isoflavones (*I*^2^ = 75.4% (95%CI = 48%–88%), *Tau*^2^ = 0.125, *p* < 0.001).

### Sensitivity analyses

**Fig 4:**
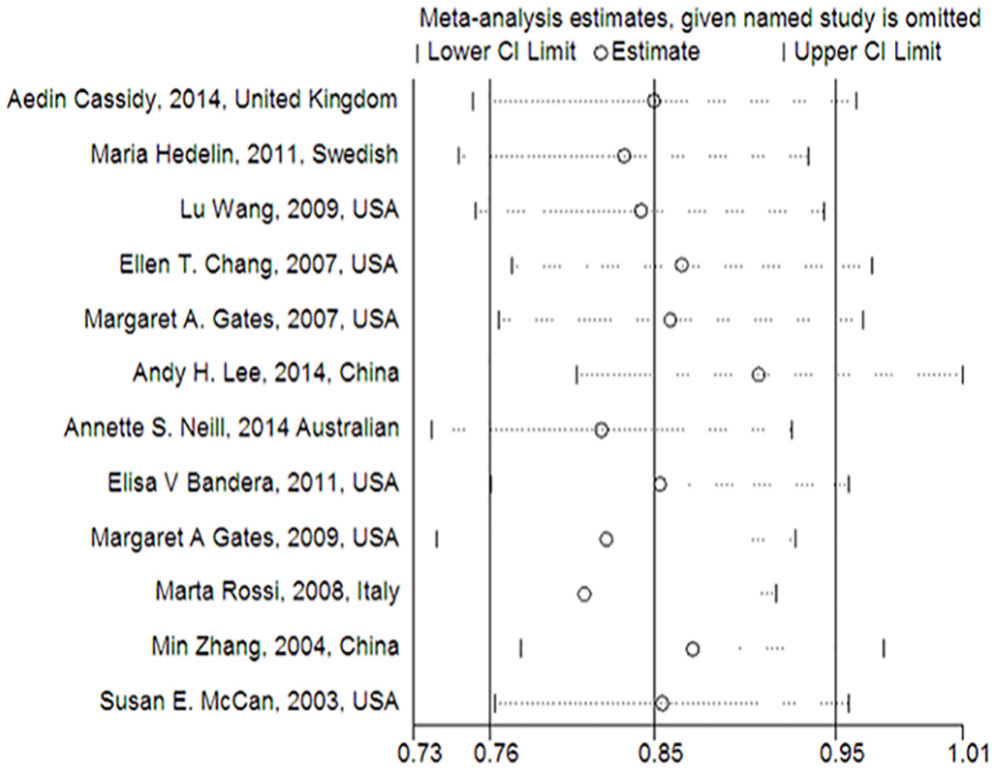
Sensitivity analysis of the overall *RR*s. (The results were calculated by omitting each eligible study. Random-effects model was used.).

A sensitivity analysis was performed to evaluate the affect of each study by sequential omission of each eligible study. The outcome revealed that the exclusion of any single study did not alter the pooled risk estimates (Fig 4). Moreover, the pooled risk estimates were not significant difference between random-effects model (*RR* = 0.82, 95% CI = 0.68–0.98) and fixed-effects model (*RR* = 0.85, 95% CI = 0.76–0.95) (Fig a in S2 File).

### Publication Bias

**Fig 5:**
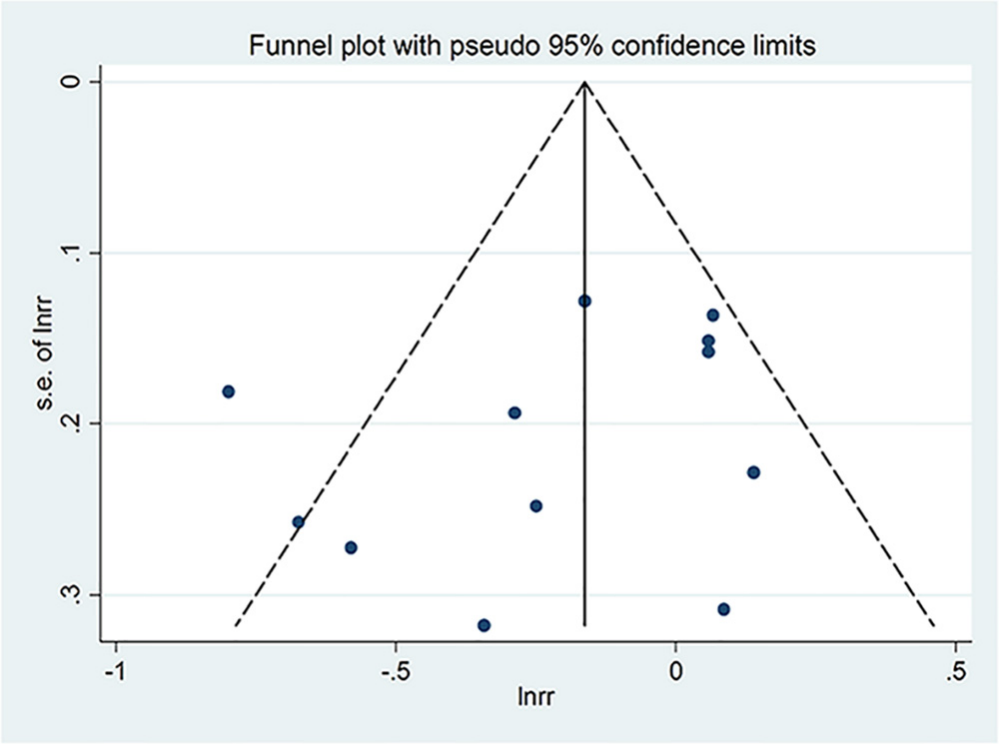
Funnel plot analysis to detect publication bias for total flavonoids.

**Fig 6:**
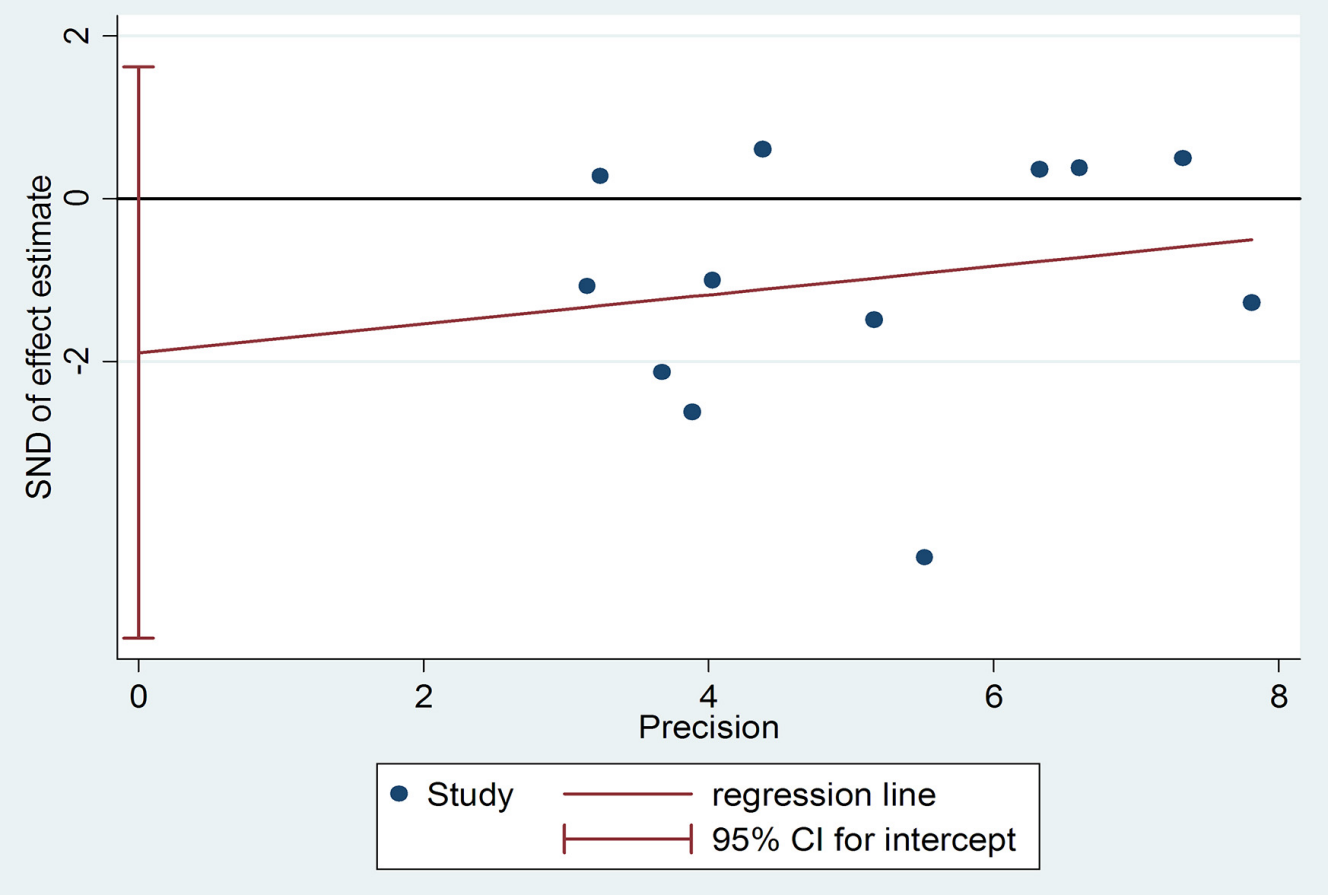
Egger’s Publication Bias Plot for total flavonoids.

Publication bias of dietary total flavonoids was evaluated with both Funnel plots and Egger’s tests. As shown in Fig 5, the shapes of the funnel plots show little evidence of publication bias among the studies. Moreover, results from Egger’s tests indicated no evidence of publication bias among these studies (*p* = 0.26). (Fig 6).

## Discussion

To the best of our knowledge, this is a comprehensive meta-analysis conducted for investigating the relationship among dietary flavonoids, flavonoid subclasses and ovarian cancer risk. The statistical analysis found that intake of dietary flavonoids can decrease ovarian cancer risk by 18%, and flavonoid subclasses: isoflavones by 33%, flavonols by 32%, respectively. That is, intake of total dietary flavonoids and their subclasses (isoflavones, flavonols) had protective effects against ovarian cancer except for flavones consumption.

At present, the influence of dietary flavonoids and flavonoid subclasses on the risk of ovarian cancer remains controversial. Some studies which were consistent with our findings have reported that dietary flavonoids or flavonoid subclasses may decrease the risk of ovarian cancer [17, 19, 36]. Although the pharmacological mechanisms underlying this effect of ovarian cancer remain unclear, it may be explained by the anticancer properties of different flavonoid subclasses [13]. There are multiple potential anticancer molecular mechanisms by which flavonoids or flavonoid subclasses might decrease the incidence of ovarian cancer [39]. Firstly, flavonoids or flavonoid subclasses could stabilize p53 gen. The stabilization of p53 often accompanies a G1 phase cell cycle arrest. Secondly, flavonoids, flavonoid subclasses might also inhibit the activity of tyrosine kinase [40]. Tyrosine kinases are a family of proteins located in or near the cell membrane involved in the transduction of growth factor signals to the nucleus. Thirdly, flavonoids are shown to modulate the inflammatory cytokines such as TNF-α and IL-6, which is inseparable with ovarian cancer. These cytokines have been shown to be involved in ovarian cancer growth and metastasis as demonstrated in various animal models and in human ovarian cancer biopsy tissues [41–43]. Fourthly, some subclasses of flavonoids have the capacity of binding with estrogen receptor, especially isoflavones [44, 45]. Therefore, flavonoids may contribute to the prevention of ovarian cancer by multiple ways.

Other studies have suggested that intake of total flavonoids may have no clear association on the incidence of ovarian cancer. Maria Hedelin's study [34] showed that no statistically significant association between ovarian cancer risk and intake of soy which are rich in isoflavones. Aedin Cassidy, et al. [12] found that total flavonoids were not statistically significantly associated with ovarian cancer risk; while participants in the highest quintiles of flavonols and flavanones intakes had modestly lower risk of ovarian cancer than participants in the lowest quintile. Several factors may explain the controversial results of these studies. Firstly, limited epidemiologic studies have evaluated the association between dietary flavonoids and ovarian cancer risk. Secondly, dietary flavonoids are widely existed in plant foods [5–7], so it is difficult to evaluate the consumption of total flavonoids or flavonoid subclasses. Thirdly, part of the controversy of epidemiological studies may be due to the difficulty in measuring intake levels of flavonoids and furthermore there were few methods for testing such as plasma metabolite levels or urinary excretion of flavonoids, which could complement dietary evaluation of the bioavailability of these dietary compounds. In summary, additional carefully designed studies should be conducted to improve the method and database for assessing dietary flavonoids, flavonoid subclasses consumption.

Several limitations should be addressed in this meta-analysis. Firstly, despite we searched all studies describing the associations of dietary flavonoids, flavonoid subclasses with ovarian cancer risk without language restricted, the number of eligible studies was still limited, especially in terms of a subgroup analysis. There are only 12 studies about total flavonoids, 4 about flavones, 7 about isoflavones, 4 about flavonols, as shown in Table 1, and the number of other subclasses such as anthocyanins and flavanones is more limited that it can not be statistical analysis. On the other hand, as the eligible studies are limited, the heterogeneity across our studies including subgroup analysis may produce biased estimates and conclusions. Previous research has shown that estimates of zero (or even low) heterogeneity should also be a concern since heterogeneity is very likely present but undetected (or underestimated). Although the bootstrapped DerSimonian-Laird leads to a small improvement over the standard random-effects model, the problem largely remains, especially for very small meta-analysis [46]. Hence, our findings should be interpreted cautiously. Secondly, as the publication year may affect publication bias [47], the selected studies in our study spanned 11 years. Therefore, potential publication bias is very likely to exist, in spite of no evidence was obtained from our statistical tests (Figs b-d in S2 File). Thirdly, different preparation and processing of food may have influenced the flavonoid levels and thus to affect the results. For example, in a recent study, orange juices were found to contain 81–200mg/L soluble flavanones, while the content in the cloud was 206–644mg/L which suggested that the flavanones are concentrated in the cloud during processing and storage [39]. However, no appropriate information was available to test this. Fourthly, accurate measurement of the average dietary flavonoids consumption is difficult, because of the wide varieties of available flavonoids and the extensive distribution in various plants. Finally, we did not retrieve the relevant published randomized controlled trial with respect to the associations among dietary flavonoids and ovarian cancer risk.

In order to explore the source of heterogeneity we conducted a meta-regression analysis in these respects of publication year, study region, cases and the NOS score (in Table 1). But none of these factors had related to estimations of effect indeed (Table a in S1 File). As mentioned above, the 12 eligible studies concerning all dietary flavonoids and flavonoid subclass, and the measurement of criteria for each flavonoid subclass was not consistent. Meanwhile, studies have suggested that different subclasses of dietary flavonoids could decrease the risk of ovarian cancer in different degrees, which also suggested that some heterogeneity exist in the study. Thus, to a certain extent, the heterogeneity across the studies was acceptable. Furthermore, in order to effectively minimize or more adequately explain heterogeneity, the methods for measuring individual consumption of dietary flavonoids and flavonoid subtypes seem also particularly desirable, as the dose of the dietary flavonoids or flavonoid subtypes might affect the protective effects regarding both patient and study results, and further higher quality studies such as well-designed especially randomized controlled trials, more comprehensive studies including more flavonoid subclass, and studies that explore the detail mechanisms of the associations among dietary flavonoids, flavonoid subclasses and ovarian cancer risk are warranted.

In contrast, there are also some advantages in our study. Our meta-analysis systematically and comprehensively sheds light on the associations among dietary flavonoids, flavonoid subclasses and ovarian cancer risk. From a public health perspective, the association between consumption of dietary flavonoids and ovarian cancer risk seems highly meaningful, as the protective effects of dietary flavonoids may provide opportunities for prevention regarding ovarian cancer. In addition, due to comprehensive analysis of more case-control and cohort studies data, our analysis increases the power and plausibility of the conclusion when compared with previous individual studies.

## Conclusions

In summary, the available evidence suggested that intake of dietary flavonoids, flavonoid subclasses (isoflavones, flavonols) has a protective effect against ovarian cancer with a reduced incidence of ovarian cancer. While the evidence for possible protection of flavones consumption against ovarian cancer was not compelling. The findings likely provide useful insight and evidence which can be used by healthcare professionals when discussing dietary flavonoids and ovarian cancer prevention with patients. While, further investigations on a larger population covering more other different flavonoid subclasses are required to confirm our findings.

## Supporting Information

S1 Table PRISMA Checklist. (DOC)

S1 File Supporting Table. (DOC)

S2 File Supporting Figures. (DOC)

## References

[1] Siegel R, Ma J, Zou Z, Jemal A.Cancer statistics, 2014. CA Cancer J Clin. 2014; 64: 9–29. 10.3322/caac.21208 24399786

[2] Ferlay J, Soerjomataram I, Dikshit R, Eser S, Mathers C, Rebelo M, Parkin DM, Forman D, Bray F. Cancer incidence and mortality worldwide: sources, methods and major patterns in GLOBOCAN 2012. Int J Cancer. 2015; 136: E359–386. 10.1002/ijc.29210 25220842

[3] American Cancer Society. Cancer Facts and Figures 2013. Atlanta American Cancer Society; 2013.

[4] Pesec M, Sherertz T.Global health from a cancer care perspective. Future Oncol. 2015; 11: 2235–2245. 10.2217/fon.15.142 26235185

[5] D'Archivio M, Filesi C, Di Benedetto R, Gargiulo R, Giovannini C, Masella R. Polyphenols, dietary sources and bioavailability.Ann Ist Super Sanita. 2007; 43: 348–361. 18209268

[6] Corcoran MP, McKay DL, Blumberg JB. Flavonoid basics: chemistry, sources, mechanisms of action, and safety.J Nutr Gerontol Geriatr. 2012; 31:176–189. 10.1080/21551197.2012.698219 22888837

[7] Ross JA, Kasum CM. Dietary flavonoids: bioavailability, metabolic effects, and safety. Annu Rev Nutr. 2002; 22: 19–34. 12055336 10.1146/annurev.nutr.22.111401.144957

[8] Kim MK, Kim JH, Nam SJ, Ryu S, Kong G. Dietary intake of soy protein and tofu in association with breast cancer risk based on a case-control study.Nutr Cancer. 2008; 60: 568–576. 10.1080/01635580801966203 18791919

[9] Hui C, Qi X, Qianyong Z, Xiaoli P, Jundong Z, Mantian M. Flavonoids, flavonoid subclasses and breast cancer risk: a meta-analysis of epidemiologic studies. PLoS One. 2013; 8: e54318 10.1371/journal.pone.0054318 23349849 PMC3548848

[10] Aune D, Lau R, Chan DS, Vieira R, Greenwood DC, Kampman E, Norat T.Nonlinear reduction in risk for colorectal cancer by fruit and vegetable intake based on meta-analysis of prospective studies. Gastroenterology. 2011; 141: 106–118. 10.1053/j.gastro.2011.04.013 21600207

[11] Tang NP, Zhou B, Wang B, Yu RB, Ma J. Flavonoids intake and risk of lung cancer: a meta-analysis. Jpn J Clin Oncol. 2009; 39: 352–359. 10.1093/jjco/hyp028 19351659

[12] Cassidy A, Huang T, Rice MS, Rimm EB, Tworoger SS.Intake of dietary flavonoids and risk of epithelial ovarian cancer.Am J Clin Nutr. 2014; 100:1344–1351. 10.3945/ajcn.114.088708 25332332 PMC4196485

[13] Kelly E. H., Anthony R. T., Dennis J. B.. Flavonoid antioxidants: chemistry, metabolism and structure-activity relationships. Journal of Nutritional Biochemistry, 2002, 13: 572–584. 12550068 10.1016/s0955-2863(02)00208-5

[14] U.S. Department of Agriculture. Iowa State University database on the isoflavone content of foods, Release 1.3. Beltsville, MD: USDA, 2002.

[15] U.S. Department of Agriculture. USDA database for the flavonoid content of selected foods. Beltsville, MD: USDA, 2003.

[16] McCann SE, Freudenheim JL, Marshall JR, Graham S. Risk of human ovarian cancer is related to dietary intake of selected nutrients, phytochemicals and food groups. J Nutr. 2003; 133: 1937–1942. 12771342 10.1093/jn/133.6.1937

[17] Zhang M, Xie X, Lee AH, Binns CW. Soy and isoflavone intake are associated with reduced risk of ovarian cancer in southeast china. Nutr Cancer. 2004; 49:125–130. 15489204 10.1207/s15327914nc4902_2

[18] Chang ET, Lee VS, Canchola AJ, Clarke CA, Purdie DM, Reynolds P, et al Diet and risk of ovarian cancer in the California Teachers Study cohort. Am J Epidemiol. 2007; 165: 802–813. 17210953 10.1093/aje/kwk065 PMC2093945

[19] Gates MA, Tworoger SS, Hecht JL, De Vivo I, Rosner B, Hankinson SE. A prospective study of dietary flavonoid intake and incidence of epithelial ovarian cancer. Int J Cancer. 2007; 121: 2225–2232. 17471564 10.1002/ijc.22790

[20] Rossi M, Negri E, Lagiou P, Talamini R, Dal Maso L, Montella M, Franceschi S, La Vecchia C. Flavonoids and ovarian cancer risk: A case-control study in Italy. Int J Cancer. 2008; 123: 895–898. 10.1002/ijc.23549 18491402

[21] Neergheen VS, Bahorun T, Taylor EW, Jen LS, Aruoma OI. Targeting specific cell signaling transduction pathways by dietary and medicinal phytochemicals in cancer chemoprevention. Toxicology. 2010; 278: 229–241. 10.1016/j.tox.2009.10.010 19850100

[22] Lee KW, Bode AM, Dong Z. Molecular targets of phytochemicals for cancer prevention. Nat Rev Cancer. 2011; 11: 211–218. 10.1038/nrc3017 21326325

[23] Vanden Berghe W.Epigenetic impact of dietary polyphenols in cancer chemoprevention: lifelong remodeling of our epigenomes. Pharmacol Res. 2012; 65:565–576. 10.1016/j.phrs.2012.03.007 22465217

[24] Dan ping S. Xiaoping W, Liqiang Q. Isoflavones Intake and Risk of Ovarian Cancer: A Meta-Analysis of Epidemiological Study. Soybean Science. 2013; 32: 814–817.

[25] Crane TE, Khulpateea BR, Alberts DS, Basen-Engquist K, Thomson CA. Dietary intake and ovarian cancer risk: a systematic review.Cancer Epidemiol Biomarkers Prev. 2014; 23: 255–273. 10.1158/1055-9965.EPI-13-0515 24142805 PMC4077283

[26] Stroup DF, Berlin JA, Morton SC, Olkin I, Williamson GD, Rennie D, et al Meta-analysis of observational studies in epidemiology: a proposal for reporting. Meta-analysis Of Observational Studies in Epidemiology (MOOSE) group. JAMA: the journal of the American Medical Association. 2000; 283(15):2008–2012. 10789670 10.1001/jama.283.15.2008

[27] Wells G, Shea B, O’Connell D, Peterson J, Welch V, Losos M, et al The Newcastle-Ottawa scale (NOS) for assessing the quality of nonrandomised studies in meta-analyses. Ottawa, ON: Ottawa Hospital Research Institute Available: www.ohri.ca/programs/clinical_epidemiology/oxford.asp. Accessed 25 October 2011.

[28] Higgins JPT and Green S (2011 Available from www.cochrane-handbook.org.) Cochrane Handbook for Systematic Reviews of Interventions Version 5.1.0 [updated March 2011]. The Cochrane Collaboration.

[29] Hardy RJ, Thompson SG. Detecting and describing heterogeneity in meta-analysis. Stat Med. 1998; 17: 841–856. 9595615 10.1002/(sici)1097-0258(19980430)17:8<841::aid-sim781>3.0.co;2-d

[30] DerSimonian R, Laird N. Meta-analysis in clinical trials. Control Clin Trials. 1986; 7: 177–188. 3802833 10.1016/0197-2456(86)90046-2

[31] Sterne Jonathan A.C, Gavaghan David, Egger Matthias. Publication and related bias in meta-analysis: Power of statistical tests and prevalence in the literature. Journal of Clinical Epidemiology. 2000; 53: 1119–1129. 11106885 10.1016/s0895-4356(00)00242-0

[32] Egger M, Davey Smith G, Schneider M, Minder C. Bias in meta-analysis detected by a simple, graphical test. BMJ. 1997; 315: 629–634. 9310563 10.1136/bmj.315.7109.629 PMC2127453

[33] Gates MA, Vitonis AF, Tworoger SS, Rosner B, Titus-Ernstoff L, Hankinson SE, Cramer DW.Flavonoid intake and ovarian cancer risk in a population-based case-control study. Int J Cancer. 2009; 124:1918–1925. 10.1002/ijc.24151 19117058 PMC2703422

[34] Hedelin M, Löf M, Andersson TM, Adlercreutz H, Weiderpass E. Dietary phytoestrogens and the risk of ovarian cancer in the women's lifestyle and health cohort study. Cancer Epidemiol Biomarkers Prev. 2011; 20:308–317. 10.1158/1055-9965.EPI-10-0752 21098648

[35] Wang L, Lee IM, Zhang SM, Blumberg JB, Buring JE, Sesso HD.Dietary intake of selected flavonols, flavones, and flavonoid-rich foods and risk of cancer in middle-aged and older women. Am J Clin Nutr. 2009; 89: 905–912. 10.3945/ajcn.2008.26913 19158208 PMC2667658

[36] Lee AH, Su D, Pasalich M, Tang L, Binns CW, Qiu L. Soy and isoflavone intake associated with reduced risk of ovarian cancer in southern Chinese women. Nutr Res. 2014; 34: 302–307. 10.1016/j.nutres.2014.02.005 24774066

[37] Neill AS, Ibiebele TI, Lahmann PH, Hughes MC, Nagle CM, Webb PM; Australian Ovarian Cancer Study Group and Australian National Endometrial Cancer Study Group.Dietary phyto-oestrogens and the risk of ovarian and endometrial cancers: findings from two Australian case-control studies. Br J Nutr. 2014; 111: 1430–1440. 24331201 10.1017/S0007114513003899

[38] Bandera EV, King M, Chandran U, Paddock LE, Rodriguez-Rodriguez L, Olson SH. Phytoestrogen consumption from foods and supplements and epithelial ovarian cancer risk: a population-based case control study. BMC Womens Health. 2011; 11: 40 10.1186/1472-6874-11-40 21943063 PMC3196697

[39] Kumar S, Pandey AK. Chemistry and biological activities of flavonoids: an overview. Scientific World Journal. 2013; 2013:162750 10.1155/2013/162750 24470791 PMC3891543

[40] Hwang KA, Park MA, Kang NH, Yi BR, Hyun SH, Jeung EB, Choi KC. Anticancer effect of genistein on BG-1 ovarian cancer growth induced by 17 β-estradiol or bisphenol A via the suppression of the crosstalk between estrogen receptor α and insulin-like growth factor-1 receptor signaling pathways.Toxicol Appl Pharmacol. 2013; 272: 637–646. 10.1016/j.taap.2013.07.027 23933164

[41] Kulbe H, Thompson R, Wilson JL, Robinson S, Hagemann T, Fatah R, Gould D, Ayhan A, Balkwill F. The inflammatory cytokine tumor necrosis factor-alpha generates an autocrine tumor-promoting network in epithelial ovarian cancer cells. Cancer Res. 2007; 67: 585–592. 17234767 10.1158/0008-5472.CAN-06-2941 PMC2679985

[42] Coward J, Kulbe H, Chakravarty P, Leader D, Vassileva V, Leinster DA, Thompson R, Schioppa T, Nemeth J, Vermeulen J, Singh N, Avril N, Cummings J, Rexhepaj E, Jirström K, Gallagher WM, Brennan DJ, McNeish IA, Balkwill FR. Interleukin-6 as a therapeutic target in human ovarian cancer. Clin Cancer Res. 2011; 17: 6083–6096. 10.1158/1078-0432.CCR-11-0945 21795409 PMC3182554

[43] Wang Y, Niu XL, Qu Y, Wu J, Zhu YQ, Sun WJ, Li LZ. Autocrine production of interleukin-6 confers cisplatin and paclitaxel resistance in ovarian cancer cells. Cancer Lett. 2010; 295: 110–123. 10.1016/j.canlet.2010.02.019 20236757

[44] Chen X, Anderson JJ. Isoflavones inhibit proliferation of ovarian cancer cells in vitro via an estrogen receptor-dependent pathway. Nutr Cancer. 2001; 41:165–171. 12094620 10.1080/01635581.2001.9680628

[45] Hwang KA, Park MA, Kang NH, Yi BR, Hyun SH, Jeung EB, Choi KC. Anticancer effect of genistein on BG-1 ovarian cancer growth induced by 17 β-estradiol or bisphenol A via the suppression of the crosstalk between estrogen receptor α and insulin-like growth factor-1 receptor signaling pathways.Toxicol Appl Pharmacol. 2013; 272: 637–646. 10.1016/j.taap.2013.07.027 23933164

[46] Kontopantelis E, Springate DA, Reeves D. A re-analysis of the Cochrane Library data: the dangers of unobserved heterogeneity in meta-analyses. PLoS One. 2013; 8: e69930 10.1371/journal.pone.0069930 23922860 PMC3724681

[47] Michal K, David A. S, Evangelos K. Publication bias in meta-analyses from the Cochrane Database of Systematic Reviews. Statist. Med. 2015; 34: 2781–2795. 10.1002/sim.6525 25988604

