# Supplementary material for: Association among Dietary Flavonoids, Flavonoid Subclasses and Ovarian Cancer Risk: A Meta-Analysis
Source: PLoS One. 2016 Mar 9;11(3):e0151134. doi: 10.1371/journal.pone.0151134 (PMC4784737; doi:10.1371/journal.pone.0151134)
Supplement: S1 Table — (DOC) [file pone.0151134.s001.doc]

| **Section/topic** | **#** | **Checklist item** | **Reported on page #** |
| --- | --- | --- | --- |
| **TITLE** | | |  |
| Title | 1 | Identify the report as a systematic review, meta-analysis, or both.  **Association among dietary flavonoids, flavonoid subclasses and ovarian cancer risk: A Meta-Analysis.** | 1 |
| **ABSTRACT** | | |  |
| Structured summary | 2 | Provide a structured summary including, as applicable: background; objectives; data sources; study eligibility criteria, participants, and interventions; study appraisal and synthesis methods; results; limitations; conclusions and implications of key findings; systematic review registration number.  **The structured summary was introduced on page 2-3 in our paper, and as our paper is a meta-analysis about the association among dietary flavonoids, flavonoid subclasses and ovarian cancer risk we have no registration number.** | 2-3 |
| **INTRODUCTION** | | |  |
| Rationale | 3 | Describe the rationale for the review in the context of what is already known.  **The details of the rationale for our meta-analysis in the context of what is already known were introduced in part of introduction in our paper.** | 3-5 |
| Objectives | 4 | Provide an explicit statement of questions being addressed with reference to participants, interventions, comparisons, outcomes, and study design (PICOS).  **Note: The PICOS structure is not applicable to this paper.** |  |
| **METHODS** | | |  |
| Protocol and registration | 5 | Indicate if a review protocol exists, if and where it can be accessed (e.g., Web address), and, if available, provide registration information including registration number.  **In our paper there was no review protocol exists.** |  |
| Eligibility criteria | 6 | Specify study characteristics (e.g., PICOS, length of follow-up) and report characteristics (e.g., years considered, language, publication status) used as criteria for eligibility, giving rationale.  **Studies were considered eligible if they met all of the following criteria: (1) the original articles described a case-control, cohort or randomized control design; (2) the article had either dietary flavonoids or subclasses of flavonoids intake as the exposure of interest; (3) the article reported the risk of ovarian cancers; and (4) the article reported 95% confidence intervals (CIs) with adjusted odds ratios (ORs) or relative risks (RRs) for ovarian cancer risk in subjects with the highest dietary flavonoid intake compared with those with the lowest dietary flavonoid intake.** | 5-6 |
| Information sources | 7 | Describe all information sources (e.g., databases with dates of coverage, contact with study authors to identify additional studies) in the search and date last searched.  **We did our best to conduct a systematic literature search in PubMed, Google Scholar and ISI Web of Science up to April 25, 2015, without language restriction.** | 5 |
| Search | 8 | Present full electronic search strategy for at least one database, including any limits used, such that it could be repeated.  **The details of search strategy was presented on page 5 in our paper** | 5 |
| Study selection | 9 | State the process for selecting studies (i.e., screening, eligibility, included in systematic review, and, if applicable, included in the meta-analysis).  **Data were extracted independently by two investigators. Studies were considered eligible if they met all of the inclusion criteria listed in our paper. If multiple articles reported the risk of ovarian cancer from the same data, the most recently published data were selected.** | 5-6 |
| Data collection process | 10 | Describe method of data extraction from reports (e.g., piloted forms, independently, in duplicate) and any processes for obtaining and confirming data from investigators.  **Data were extracted independently by two investigators, according to the inclusion and exclusion criteria. The following data: first author, publication year, study region, study design, data acquisition approach, number of cases and controls, types and consumption of flavonoids, controlled confounders adjusted for in multivariate analysis, OR or RR and 95% CI were collected. The quality of each study was evaluated using the Newcastle-Ottawa Scale (NOS) quality assessment criteria. The quality scores of the studies ranged from 0 to 9.0, Scores＜7.0 indicated low quality, while those ≥7.0 indicated high quality.** | 6 |
| Data items | 11 | List and define all variables for which data were sought (e.g., PICOS, funding sources) and any assumptions and simplifications made.  **The data was listed in table 1.** | 9-11 |
| Risk of bias in individual studies | 12 | Describe methods used for assessing risk of bias of individual studies (including specification of whether this was done at the study or outcome level), and how this information is to be used in any data synthesis.  **Sensitivity analysis was performed to assess the robustness of the results of the combined effects, which were performed by sequential removal of each study.** | 7 |
| Summary measures | 13 | State the principal summary measures (e.g., risk ratio, difference in means).  **The principal summary measure is risk ratio (RR).** | 6 |
| Synthesis of results | 14 | Describe the methods of handling data and combining results of studies, if done, including measures of consistency (e.g., I^2^) for each meta-analysis.  **The pooled RR were computed by the adjusted RRs or ORs and 95% CIs reported in the studies. The ORs were considered to correspond to RRs. Cochran Q statistic and I^2^ were used for the assessment of heterogeneity across the studies. Nevertherless, in view of the limitations of Cochran Q, especially for small meta-analysis, Tau^2^ was also provided. In addition, a random effects model described by DerSimonian-Laird method was preferred to calculate the summarized estimates and corresponding 95% CIs.** | 7 |

Page 1 of 2

| **Section/topic** | **#** | **Checklist item** | **Reported on page #** |
| --- | --- | --- | --- |
| Risk of bias across studies | 15 | Specify any assessment of risk of bias that may affect the cumulative evidence (e.g., publication bias, selective reporting within studies).  **Publication bias was assessed with Funnel plots and Egger’s tests.** | 7 |
| Additional analyses | 16 | Describe methods of additional analyses (e.g., sensitivity or subgroup analyses, meta-regression), if done, indicating which were pre-specified.  **A sensitivity analysis was performed to evaluate the affect of each study by sequential omission of each eligible study. The outcome revealed that the exclusion of any single study did not alter the pooled risk estimates (Fig 4). Moreover, the pooled risk estimates were not significant difference between random-effects model (RR =0.82, 95% CI=0.68-0.98) and fixed-effects model (RR=0.85, 95% CI=0.76-0.95)** | 14 |
| **RESULTS** | | |  |
| Study selection | 17 | Give numbers of studies screened, assessed for eligibility, and included in the review, with reasons for exclusions at each stage, ideally with a flow diagram.  **A total of 68 articles were identified in the initial search. Of these articles, 46 were excluded after reviewing the titles and abstracts, removing duplicates. Then, by thoroughly reading the full text 10 articles were also excluded because they did not provide information about flavonoids or flavonoid subclasses intake, ovarian cancer risk, or 95% CI. Finally, a total of 12 articles met the inclusion criteria and were included in the final meta-analysis.**  **The flow diagram was shown in Fig 2.** | 7-8 |
| Study characteristics | 18 | For each study, present characteristics for which data were extracted (e.g., study size, PICOS, follow-up period) and provide the citations.  **The characteristics of each study were presented in table 1.** | 9-11 |
| Risk of bias within studies | 19 | Present data on risk of bias of each study and, if available, any outcome level assessment (see item 12).  **As shown in Fig 5, the shapes of the funnel plots show little evidence of publication bias among the studies. Moreover, results from Egger’s tests indicated no evidence of publication bias among these studies (p = 0.26).** | 13 |
| Results of individual studies | 20 | For all outcomes considered (benefits or harms), present, for each study: (a) simple summary data for each intervention group (b) effect estimates and confidence intervals, ideally with a forest plot.  **The outcomes of each study were shown in Fig.3.** | 12 |
| Synthesis of results | 21 | Present results of each meta-analysis done, including confidence intervals and measures of consistency.  **The meta-analysis of the five cohort studies and seven case-control studies indicated that ovarian cancer risk was significantly reduced (RR =0.82, 95% CI=0.68-0.98) in women with highest intakes of total flavonoids, compared with that in those with lowest intakes of total flavonoids. That is, consumption of dietary flavonoids has a protective effect against ovarian cancer. The heterogeneity among the studies was significant (I^2^ = 62% (95%CI = 28% - 80%), Tau^2^ = 0.059, p = 0.002) from a random effect model (Fig 3 A).** | 12,13 |
| Risk of bias across studies | 22 | Present results of any assessment of risk of bias across studies (see Item 15).  **The results of any assessment of risk of bias across studies were shown in Fig.5 and Fig.6** | 13 |
| Additional analysis | 23 | Give results of additional analyses, if done (e.g., sensitivity or subgroup analyses, meta-regression [see Item 16]).  **The results of the subgroup meta-analysis and sensitivity analysis were shown on paper12 and13.** | 12,13 |
| **DISCUSSION** | | |  |
| Summary of evidence | 24 | Summarize the main findings including the strength of evidence for each main outcome; consider their relevance to key groups (e.g., healthcare providers, users, and policy makers).  **To the best of our knowledge, this is a comprehensive meta-analysis conducted for investigating the relationship among dietary flavonoids, flavonoid subclasses and ovarian cancer risk. The statistical analysis found that intake of dietary flavonoids can decrease ovarian cancer risk by 18%, and flavonoid subclasses: isoflavones by 33%, flavonols by 32%, respectively. That is, intake of total dietary flavonoids and their subclasses (isoflavones, flavonols) had protective effects against ovarian cancer except for flavones consumption.** | 13,14 |
| Limitations | 25 | Discuss limitations at study and outcome level (e.g., risk of bias), and at review-level (e.g., incomplete retrieval of identified research, reporting bias).  **The limitations at study and outcome level and at review-level were discussed on page 15-16 in our paper.** | 15,16 |
| Conclusions | 26 | Provide a general interpretation of the results in the context of other evidence, and implications for future research.  **The available evidence suggested that intake of dietary flavonoids, flavonoid subclasses (isoflavones, flavonols) has a protective effect against ovarian cancer with a reduced incidence of ovarian cancer. While the evidence for possible protection of flavones consumption against ovarian cancer was not compelling.** | 17 |
| **FUNDING** | | |  |
| Funding | 27 | Describe sources of funding for the systematic review and other support (e.g., supply of data); role of funders for the systematic review.  **This study was supported by the research grant from National Natural Science Foundation of China (81372807 and 81503427) and Natural Science Foundation of Hubei Province (2015CKB751).** | 18 |

*From:*  Moher D, Liberati A, Tetzlaff J, Altman DG, The PRISMA Group (2009). Preferred Reporting Items for Systematic Reviews and Meta-Analyses: The PRISMA Statement. PLoS Med 6(6): e1000097. doi:10.1371/journal.pmed1000097

For more information, visit: **www.prisma-statement.org**.

Page 2 of 2
