# Supplementary material for: Association among Dietary Flavonoids, Flavonoid Subclasses and Ovarian Cancer Risk: A Meta-Analysis
Source: PLoS One. 2016 Mar 9;11(3):e0151134. doi: 10.1371/journal.pone.0151134 (PMC4784737; doi:10.1371/journal.pone.0151134)
Supplement: S1 File — (DOC) [file pone.0151134.s002.doc]

**Table a. The result of meta-regression analysis in respects of publication year, study region, score and number of cases.**

| ln*rr* | Coef. | Std. Err. | *t* | *p*>|*t*| | [95% Conf. Interval] | |
| --- | --- | --- | --- | --- | --- | --- |
| Publication year | -0.034922 | 0.0508719 | -0.69 | 0.515 | -0.155215 | 0.0853714 |
| study region | -0.063297 | 0.1187605 | -0.53 | 0.611 | -0.344121 | 0.2175268 |
| score | 0.3024853 | 0.231056 | 1.31 | 0.232 | -0.243875 | 0.8488459 |
| number of cases | -8.65E-07 | 2.73E-06 | -0.32 | 0.761 | -7.33E-06 | 5.60E-06 |
| _cons | 68.01038 | 101.0286 | 0.67 | 0.522 | -170.8843 | 306.905 |
