# Supplementary material for: Association among Dietary Flavonoids, Flavonoid Subclasses and Ovarian Cancer Risk: A Meta-Analysis
Source: PLoS One. 2016 Mar 9;11(3):e0151134. doi: 10.1371/journal.pone.0151134 (PMC4784737; doi:10.1371/journal.pone.0151134)
Supplement: S2 File — (DOC) [file pone.0151134.s003.doc]

**Fig a. Forest plot describing the association between dietary flavonoids intake and ovarian cancer risk (fixed effects model).**

**Fig b. Egger’s Publication Bias Plot for flavones. （*p* = 0.955）**

**Fig c. Egger’s Publication Bias Plot for Isoflavones ( *p*=0.837).**

**Fig d. Egger’s Publication Bias Plot for Flavonols ( *p* = 0.794).**
